# Sensitivity to Interaural Phase in Older Hearing-Impaired Listeners Correlates With Nonauditory Trail Making Scores and With a Spatial Auditory Task of Unrelated Peripheral Origin

**DOI:** 10.1177/2331216519864499

**Published:** 2019-08-28

**Authors:** Olaf Strelcyk, Pavel Zahorik, James Shehorn, Chhayakanta Patro, Ralph Peter Derleth

**Affiliations:** 1 Sonova U.S. Corporate Services, Warrenville, IL, USA; 2 Department of Otolaryngology and Communicative Disorders, University of Louisville, Louisville, KY, USA; 3 Department of Psychological and Brain Sciences, University of Louisville, Louisville, KY, USA; 4 Heuser Hearing Research Center, Louisville, KY, USA; 5 Sonova AG, Stäfa, Switzerland

**Keywords:** hearing loss, interaural level, interaural phase, older listeners, trail making

## Abstract

Interaural phase difference (IPD) discrimination upper frequency limits and just-noticeable differences (JNDs), interaural level difference (ILD) JNDs, and diotic intensity JNDs were measured for 20 older hearing-impaired listeners with matched moderate sloping to severe sensorineural hearing losses. The JNDs were measured using tone stimuli at 500 Hz. In addition to these auditory tests, the participants completed a cognitive test (Trail Making Test). Significant performance improvements in IPD discrimination were observed across test sessions. Strong correlations were found between IPD and ILD discrimination performance. Very strong correlations were observed between IPD discrimination and Trail Making performance as well as strong correlations between ILD discrimination and Trail Making performance. These relationships indicate that interindividual variability in IPD discrimination performance did not exclusively reflect deficits specific to any auditory processing, including early auditory processing of temporal information. The observed relationships between spatial audition and cognition may instead be attributable to a modality-general spatial processing deficit and/or individual differences in global processing speed.

## Introduction

The discrimination of interaural time differences (ITDs) and interaural phase differences (IPDs) are suprathreshold functions of binaural hearing that facilitate the localization of sound sources in the horizontal plane. Hearing-impaired (HI) listeners can show poorer than normal ITD and IPD discrimination performance even in the absence of a pure-tone hearing loss at the test frequencies (e.g., Koehnke, Culotta, Hawley, & Colburn, 1995; Moore, Glasberg, Stoev, Füllgrabe, & Hopkins, 2012; Neher, Laugesen, Jensen, & Kragelund, 2011; Smoski & Trahiotis, 1986; Strelcyk & Dau, 2009). For this reason, IPD discrimination has recently been employed in studies searching for cochlear synaptopathy in humans by exploring the presence of functional impairments in noise-exposed individuals with clinically normal audiograms (Grose, Buss, & Hall, 2017; Prendergast et al., 2017).

Interindividual variability in IPD discrimination performance among HI listeners has been found to be, at best, moderately associated with pure-tone hearing thresholds (Füllgrabe & Moore, 2017, 2018; Füllgrabe, Sek, & Moore, 2018; King et al., 2014; Moore & Sek, 2016). Furthermore, weak to moderate correlations with listener age have been reported (Füllgrabe & Moore, 2018; Füllgrabe et al., 2018; King et al., 2014; Neher et al., 2011; Neher, Lunner, Hopkins, & Moore, 2012; Ross, Fujioka, Tremblay, & Picton, 2007a). Beginning in mid-life, IPD discrimination performance tends to decrease with age (Füllgrabe, 2013; Grose & Mamo, 2010; Ross et al., 2007a). In a meta-analysis of 19 previous studies, Füllgrabe and Moore (2018) concluded that the percentage of variance in IPD discrimination performance accounted for by the combination of age and hearing thresholds ranged from 8% to 42%, depending on test frequency, leaving substantial amounts of interindividual performance variability unexplained. Since IPD sensitivity is not fully predicted by pure-tone hearing thresholds and can degrade with aging, IPD discrimination may complement traditional audiologic measures in building comprehensive auditory profiles beyond the audiogram (e.g., Houtgast & Festen, 2008; Sanchez Lopez, Bianchi, Fereczkowski, Santurette, & Dau, 2018; Thorup et al., 2016), particularly given that IPD discrimination performance has been shown to be associated with real-world outcomes such as speech perception performance in the presence of spatially separated interferers (Füllgrabe, Moore, & Stone, 2015; Neher et al., 2011, 2012; Oberfeld & Klöckner-Nowotny, 2016; Papesh, Folmer, & Gallun, 2017; Strelcyk & Dau, 2009).

At the level of the basilar membrane, sound signals can be analyzed in terms of an envelope imposed on a rapidly oscillating carrier, referred to as temporal fine structure (TFS; Moore, 2008). Information in the TFS is encoded neurally by phase locking: precisely timed action potentials in the auditory nerve (McAlpine, 2005; Young & Oertel, 2004). IPD discrimination requires a binaural decoding of TFS information. Under the assumption that TFS sensitivity, that is, the auditory system’s capacity for processing TFS information, is the main determinant of IPD discrimination performance and that other factors such as nonauditory processing efficiency or cognitive abilities are of minor influence, IPD discrimination has commonly been regarded as a measure of TFS sensitivity (e.g., Füllgrabe, Harland, Sek, & Moore, 2017; Füllgrabe et al., 2018; Hopkins & Moore, 2010; Lacher-Fougère & Demany, 2005; Strelcyk & Dau, 2009). However, some studies have found IPD discrimination performance by older NH and HI listeners to be significantly associated with cognitive abilities (Füllgrabe et al., 2015; Neher et al., 2012; Rönnberg et al., 2016). Thus, it remains to be clarified if or under which conditions IPD discrimination performance may be affected by factors other than TFS sensitivity per se.

The idea for this study arose from observations we made in a previous, unpublished study. Among other psychoacoustic tests in that study, we measured IPD discrimination upper frequency limits (FLs) using tone sequence stimuli similar to those used by Neher et al. (2011) but with a raised cosine instead of a fully rectified sinusoid as envelope modulator. Four of the 20 older participants with moderate low-frequency hearing losses were not able to perform the IPD discrimination task above chance. We conducted follow-up sessions with three of the participants who failed to perform the task (the fourth participant was not available), in order to familiarize them further with the IPD stimuli and to give them task-specific training. However, they still were not able to detect IPDs at any frequency down to 125 Hz. Furthermore, they were not able to discriminate interaural level differences (ILDs) at 500 Hz either. We also administered a Montreal Cognitive Assessment (MoCA; Lin et al., 2017). Although there was no indication of mild cognitive impairments based on their MoCA scores, we observed that two of the three participants failed on the first MoCA task, an adapted, untimed Trail Making B task (Reitan, 1955), which involved drawing a line connecting 10 alternating numbers and letters in sequence (1-A-2-B and so on; participants earned one point in this MoCA task if they successfully completed the trail and failed if, in drawing the line, they made an error that was not immediately self-corrected). Based on these observations, we conducted this study to test the hypothesis that IPD discrimination performance is associated with performance in other tasks, such as ILD discrimination and Trail Making, in a sample of older HI listeners with matched audiograms.

We measured IPD discrimination FLs and just-noticeable differences (JNDs) repeatedly across test sessions to explore potential training effects. Furthermore, we included intensity discrimination and ILD discrimination tasks to examine whether listeners who show difficulties with IPD discrimination would also experience difficulties with these tasks as suggested by previous studies (Ochi, Yamasoba, & Furukawa, 2016; Spencer, Hawley, & Colburn, 2016; Whiteford, Kreft, & Oxenham, 2017). In addition, we investigated potential relationships between interaural discrimination performance and cognitive abilities in terms of Trail Making performance. The Trail Making Test part A (TMA), which involved tapping numbers in sequence on a touchscreen (1–2–3–4 and so on), indexed processing speed, visual search, and motor skills. In addition, the Trail Making Test part B (TMB), which involved tapping alternating numbers and letters in sequence (1–A–2–B and so on), required executive control abilities such as manipulating information in working memory and attentional task-switching ability (Arbuthnott & Frank, 2000; Bowie & Harvey, 2006; Sánchez-Cubillo et al., 2009). The inclusion of the Trail Making Test was partly motivated by Woods, Kalluri, Pentony, and Nooraei (2013), who observed that TMB time accounted for variability in speech perception performance among HI listeners in the presence of spatially separated interferers after individual differences in audibility were accounted for. Similarly, Füllgrabe et al. (2015) reported a significant correlation between TMB time and sentence identification in spatially separated interferers in a group of older NH listeners. Previous studies that measured IPD discrimination using tone sequence stimuli mostly included HI listeners with slight to mild hearing losses in the low frequencies (e.g., Füllgrabe & Moore, 2017; Hopkins & Moore, 2011; King et al., 2014; Moore & Sek, 2016; Neher et al., 2011, 2012; Santurette & Dau, 2012). In contrast, our participants had moderate losses in the low frequencies. They had very similar audiometric profiles that fell within the range between the N3 and N4 standard audiograms, which are most frequently encountered in clinical practice (Bisgaard, Vlaming, & Dahlquist, 2010). Furthermore, previous studies used adaptive staircase methods to estimate upper FLs of IPD discrimination whereas we used a Bayesian procedure (Remus & Collins, 2008). In particular, this procedure allowed for efficient detection of chance performance, which can be a problem with staircase methods (cf. Bianchi, Carney, Dau, & Santurette, 2019).

## Methods

### Participants

The 20 HI participants (11 women and nine men) were aged between 48 and 85 years (median: 71 years). Their demographic and audiologic information is detailed in Table 1. They had bilaterally symmetric audiograms with ear asymmetries equal to or less than 10 dB at all octave frequencies from 125 to 8000 Hz and 750 to 6000 Hz (exceptions are stated in the table). Pure-tone thresholds averaged across 500, 1000, 2000, and 4000 Hz ranged from 43 to 58 dB hearing level (HL, American National Standards Institute, 2018), while low-frequency pure-tone thresholds, averaged across all frequencies from 125 to 1500 Hz (PTA_LF_), ranged from 37 to 52 dB HL. All ears showed clear ear canals under otoscopic inspection, and air-bone gaps were smaller than or equal to 10 dB, except for participants f9 and m2. Participant f9 showed an air-bone gap of 15 dB at 500 Hz in the right ear, and m2 showed air-bone gaps of 20 dB at 125 and 250 Hz in both ears. Furthermore, m2 was the only participant who reported having had ear tubes inserted at age 8 years. Thus, participants f9 and m2 may have had mixed hearing losses with small conductive components in the low frequencies, while the remaining participants showed hearing losses of purely sensorineural origin. Table 1 lists the following additional characteristics based on self-report: The age at which the participant’s hearing loss was detected, whether they experienced tinnitus at least sometimes or not, and how many years of one-on-one musical training they had received. All participants were native speakers of American English and participated in psychoacoustic measurements for the first time. Eighteen of the 20 participants were experienced hearing-aid (HA) users, who had been wearing HAs for more than 1 year, while participant f4 had been wearing HAs for 3 months and participant f9 did not use HAs at the time when this study took place.

**Table 1.** Age (years), Audiometric Thresholds (dB HL), Age When Hearing Loss Was Detected (years), Presence of Tinnitus, Years of Formal Music Education, and MoCA Score for the 20 HI Participants.

		Pure-tone audiometric thresholds, left ear/right ear											Age detected	Tinnitus	Music education	MoCA
ID	Age	125	250	500	750	1000	1500	2000	3000	4000	6000	8000				
f1	48	35/35	40/40	45/45	50/50	50/50	50/55	60/60	60/60	65/65	75/75	80/75	8	Yes	0	24
m2	57	60/60	60/60	50/50	35/40	40/40	55/50	60/55	60/60	70/65	90/85	95/95	8	No	0	28
m3	61	35/35	35/35	40/40	55/55	60/55	65/65	65/65	65/60	65/60	70/65	70/65	45	No	10	29
f4	65	nm	35/40	35/40	nm	35/35	nm	45/45	55/55	60/45 ^a^	65/65	75/75	64	Yes	4	28
f5	66	50/45	45/45	45/45	50/45	55/45	55/45	50/50	50/50	60/55	70/75	80/80	40	Yes	0	24
f6	66	40/35	40/40	40/40	40/45	40/45	45/45	50/45	55/55	60/60	70/75	75/70	64	No	0	29
f7	68	40/40	50/50	55/50	55/55	55/55	60/55	60/55	60/60	60/60	65/65	70/70	58	No	0	26
f8	68	35/45	35/45	50/45	50/55	60/60	60/60	60/65	55/65	55/60	55/65	55/85 ^a^	50	Yes	0	27
f9	69	40/45	35/45	40/35	40/40	45/40	50/40	55/50	65/65	65/60	65/65	80/75	67	No	1	26
f10	70	40/50	40/50	40/45	55/50	55/50	55/60	60/55	55/65	60/65	65/65	70/75	55	No	11	28
f11	71	30/30	30/30	40/35	40/40	55/50	65/55	65/55	65/55	60/55	65/60	60/60	60	No	10	28
m12	71	30/40	35/40	40/45	40/40	45/45	65/65	70/65	70/65	75/75	70/65	65/70	61	Yes	8	24
f13	74	40/35	40/35	50/45	55/50	55/50	55/50	60/55	65/65	65/60	75/65	90/80	8	Yes	0	27
m14	75	30/30	30/35	35/40	40/45	40/45	45/45	50/50	50/45	60/50	70/60	70/75	50	No	0	24
m15	76	30/35	40/40	40/40	45/45	50/50	50/50	60/60	60/55	70/65	65/70	75/75	21	Yes	0	29
m16	76	25/35	35/40	40/40	40/40	40/50	45/50	55/60	70/80	80/85	80/80	90/95	60	Yes	0	23
f17	78	30/30	30/30	40/35	40/40	45/45	45/50	45/45	55/45	65/60	70/70	80/80	65	Yes	0	25
m18	80	40/35	40/35	35/35	40/40	45/45	60/60	60/65	60/65	65/70	60/65	55/60	76	No	10	22
m19	81	20/25	25/25	40/35	50/50	45/50	50/55	50/50	50/50	55/60	50/50	70/60	74	No	0	24
m20	85	35/30	35/30	40/40	45/45	45/45	45/50	45/50	50/60	55/55	55/55	70/55 ^a^	71	Yes	2	24
**Mean**	**70**	**36/38**	**38/40**	**42/41**	**46/46**	**48/48**	**54/53**	**56/55**	**59/59**	**64/62**	**68/67**	**74/74**				**26.0**

*Note*. The first letter of the ID indicates gender (female and male). nm = not measured.

^a^Left/right asymmetry larger than 10 dB.

Participants were paid $20 per hour. The study was approved by the Biomedical Institutional Review Board of the University of Louisville.

### Cognitive Tests

#### Montreal Cognitive Assessment

A version of the MoCA for the hearing impaired with visual instructions was administered nonverbally using a timed PowerPoint (Microsoft Corp.) presentation (Lin et al., 2017). The MoCA tested performance on a variety of tasks such as TMB, clock drawing, animal naming, word recall, or serial subtraction assessing executive function, visuospatial skills, verbal fluency, language, attention, abstraction, and orientation. Therefore, the overall test score was used as a global measure of cognitive performance. The scores were corrected for education effects by adding one point for participants f6, f8, and m19, who had 12 or fewer years of formal education (Nasreddine et al., 2005). Participant m18 showed the lowest score with a value of 22 out of 30. Based on a cutoff score of 23 (Carson, Leach, & Murphy, 2018), his score may indicate a mild cognitive impairment. However, since our objective was to explore relations between cognitive and psychoacoustic abilities and this participant showed no difficulty with performing all tasks, we decided to include his data in the analyses.

#### Trail Making Test

Part A of the Trail Making Test (connecting 25 numbers in sequence, 1–2–3–4 …) (Reitan, 1955) was administered to assess processing speed, visual search and motor skills. Part B (connecting 25 alternating numbers and letters in sequence, 1–A–2–B…) was administered to capture executive function in addition to these cognitive abilities (e.g., Arbuthnott & Frank, 2000; Bowie & Harvey, 2006; Sánchez-Cubillo et al., 2009). The test was implemented in the Psychology Experiment Building Language (PEBL version 2.0.4; Mueller & Piper, 2014) and performed via a touchscreen display (GeChic 1303i 13.3-in.). It was based on the PEBL Trail Making Test (Piper et al., 2012) but modified as described in the following. Similar to paper-pencil versions of the tests, 25 black circles enclosing numbers or letters were displayed on a white background. In the TMA test, we placed the circles in the layout of the Halstead-Reitan test, part A (Reitan, 1955), albeit scaled to the landscape format of the touchscreen (1,920 × 1,080 pixels). For the TMB test, the layout was mirrored horizontally to preserve the distances and relative arrangements of the circles in the TMA test. Participants were instructed to complete the trails as quickly as possible. The instructions were given nonverbally, as detailed in Appendix A. When a participant touched the correct circle, the circle briefly lit up in green and a black line segment was drawn from the previous circle to the present circle. When they touched the wrong circle, the circle briefly lit up in red and no connecting line segment was drawn.

Participants first performed a TMA practice run on a shorter layout consisting of eight circles before performing the 25-circle TMA test. Subsequently, they performed a short practice run of TMB followed by the full 25-circle TMB test. In contrast to the MoCA Trail Making subtask, the Trail Making Test was timed, and the test score was the time to complete the trail starting from when the circles were first displayed on the screen. Participants could only fail the TMA or TMB tests if they were not able to complete each trail within the allowed maximum time of 300 seconds, at which point the tests were automatically aborted (cf. Bowie & Harvey, 2006).

In addition to the TMA and TMB times, we calculated the difference score TMB–TMA, which has been considered to be an indicator of executive control abilities (Sánchez-Cubillo et al., 2009).

### Psychoacoustic Tests

#### IPD discrimination upper FL

We used a two-interval, two-alternative, forced-choice (2I-2AFC) task similar to the TFS test described by Füllgrabe et al. (2017) to measure IPD discrimination upper FLs. Each trial consisted of two intervals separated by 400 ms. Each interval contained four 400-ms tones (including 50-ms raised-cosine onset/offset ramps) separated by 25-ms silent gaps. All tones in the reference interval had 0° IPDs. In the randomly chosen target interval, the first and third tones had 0° IPDs, while the second and fourth tones had 180° IPDs (−90° and 90° phase shifts in left and right ears, respectively). Normal-hearing listeners would perceive these antiphasic tones as lateralized to one side or as diffusely localized in the ears (Füllgrabe et al., 2017; Kunov & Abel, 1981). The frequency of the tones was varied adaptively from trial to trial. At any given frequency, the tones were presented at 30 dB sensation level (SL; estimated from audiometric thresholds as described in the Apparatus subsection below) unless this would have resulted in a presentation level of more than 95 dB A-weighted sound pressure level (dBA) at the average human ear drum, which was simulated using ear simulators (GRAS RA0045) in a KEMAR manikin (GRAS 45BB-7; Burkhard & Sachs, 1975) with anthropometric pinnae (GRAS KB5000, KB5001). In these cases, the presentation level was lowered to 95 dBA. This yielded presentation levels of 30 dB SL for all participants but m3, f8, and f11, for whom the minimum presentation levels were 15, 20, and 20 dB SL, respectively (in all three cases, these minimum presentation levels were reached at 1350 Hz or higher; up to 1000 Hz, all presentation levels exceeded 25 dB SL).

Participants were instructed to identify the interval in which the sounds were perceived to move (see Appendix A for detailed participant instructions). The intervals were visually marked during playback by flashing boxes on a touchscreen display (GeChic 1002 10.1-in.) placed in front of the participants, and they responded by touching the corresponding box. Tone frequency was varied adaptively from trial to trial using the Bayes Fisher information gain method described by Remus & Collins (2008). The set of possible tone frequencies were 57 logarithmically scaled values from 125 to 2000 Hz, with the exception of the initial frequency which was 500 Hz in all runs.

Similar to the procedures in Bianchi et al. (2019) and Füllgrabe et al. (2017), each IPD discrimination measurement was preceded by a training run with IPD cues replaced by ILD cues. This ILD training run consisted of 15 trials, in which the movement in the target interval was induced by introducing ILDs rather than 180° IPDs in the second and fourth tones. The ILDs were adaptively varied (Remus & Collins, 2008), while tone frequency was fixed at 500 Hz. To prevent the participants from learning loudness rather than movement cues, the levels of the second and fourth tones in the reference interval were increased by ILD/2 in both ears to match the levels of the tones in the target interval in the right ear, which had the level increment of ILD/2. Subsequently, the participants performed a 10-trial training run of the actual IPD discrimination task with adaptively varied tone frequency, followed by the measurement run consisting of 60 trials. Correct-response feedback was provided throughout all training and measurement runs (this applies to all psychoacoustic tests in this study).

For each 60-trial measurement run, IPD discrimination performance as a function of log-transformed tone frequency was modeled as a logistic psychometric function (PF) with negative slope and fitted using the *psignifit 4* MATLAB toolbox for Bayesian PF estimation (Schütt, Harmerling, Macke, & Wichmann, 2016). The IPD FL was defined as the tone frequency corresponding to the 75 percent-correct point on the estimated PF. Since the lowest test frequency was limited to 125 Hz, the fitted IPD FL would have been larger than 0 Hz even in the case of random responses. To determine the specificity of our procedure to detect chance performance, we simulated 2,000 random-response measurement runs: 98% of the fitted IPD FLs fell below 125 Hz (note that the same result was obtained when the simulations were repeated with a start frequency of 250 Hz instead of 500 Hz). Thus, if a participant’s IPD FL was estimated to be greater than 125 Hz, we could infer with 98% confidence that the participant had not been guessing randomly. To determine the sensitivity of our procedure to detect near-chance performance, we ran simulations for a series of poorly performing observers with varying PF thresholds (IPD FLs) and widths. For an observer with a true IPD FL of 100 Hz and a shallow PF slope (PF spanning six octaves from the 53 to 97 percent-correct points, which was the shallowest slope observed in this study), only 5% of fitted FLs fell below 65 Hz. The percentage was lower for observers with higher true FLs and/or steeper PFs. Thus, if a participant’s fitted IPD FL fell below 65 Hz, we could infer with 95% confidence that their true FL was not larger than 100 Hz (assuming their true PF width did not exceed six octaves).

#### Interaural phase difference JND

The 2I-2AFC measurement of IPD JND was very similar to the measurement of IPD discrimination FL. The main difference in the IPD JND measurement was that the tone frequency was fixed at 500 Hz, while the IPDs of the second and fourth tones in the target interval were varied adaptively to measure discrimination performance as a function of IPD. The IPDs of the second and fourth tones had opposite signs, such that the second tone was lateralized toward the left and the fourth tone was lateralized toward the right side. This was done to provide participants with movement cues to both sides, since the side to which the target tones with 180° IPDs in the IPD FL measurement were lateralized was ambiguous. The set of possible IPDs in the adaptive run (Remus & Collins, 2008) were 50 logarithmically scaled values from 0.5° to 180°, with the exception of the initial IPD which was 90° in all runs. The presentation level for all participants was 30 dB SL. The instructions were the same as for the measurement of IPD discrimination FL.

Participants performed a training run consisting of 10 trials before performing a measurement run of 60 trials. In contrast to the measurements of IPD discrimination FL, ILD training runs were not included in the IPD JND measurements. IPD discrimination performance as a function of log-transformed IPD was fitted using a logistic PF model (Schütt et al., 2016), and the IPD JND was defined as the IPD corresponding to the 75 percent-correct point.

#### Interaural level difference JND

Similar to the IPD JND measurement, the ILD JND measurement was also performed at a fixed frequency of 500 Hz. The main difference was that ILDs were used to induce movement instead of IPDs. In the reference interval, all tones had a 0-dB ILD. In the target interval, the first and third tones also had 0-dB ILDs, while the second and fourth tones had a finite ILD that was varied adaptively. The ILD was applied by lowering the tone level on the left by ILD/2 and by increasing it on the right by ILD/2. Thus, both the second and fourth tones were lateralized toward the right. The set of possible ILDs in the adaptive run (Remus & Collins, 2008) were 80 logarithmically scaled values from 0.1 to 16 dB, with the exception of the initial ILD which was 6 dB in all runs.

ILD JNDs were measured in three different experimental conditions similar to those used by L. R. Bernstein (2004). There was a No Rove condition with the ILDs applied as described above (all four tones in the reference interval had identical levels). Furthermore, there was a Level Rove condition, in which the levels of all four tones in both the reference and target intervals were roved independently to weaken nuisance single-ear cues such as loudness differences. Finally, in an IPD/Level Rove condition, random IPDs were applied to the second and fourth tones in both the reference and target intervals in addition to roving the individual tone levels. The IPDs of the second and fourth tones had opposite signs, such that they would shift lateralization of the second and fourth tone to the left and right, respectively. The IPD roving was added to explore whether random IPD cues would interfere with ILD discrimination. The level adjustments in the Level Rove and IPD/Level Rove conditions were chosen randomly from −10 to 10 dB (in 0.5-dB steps) for each trial and each of the eight tones. The absolute values of the IPDs in the IPD Rove condition were chosen randomly from 0° to 45° (in 1° steps) for each trial and each of the four second and fourth tones. For large enough ILDs, the roving applied in the Level Rove condition would not have prevented participants from performing the task based on a loudness comparison between successive tones. In accordance with Green (1988), the threshold ILD beyond which participants could have relied solely on single-ear cues was 11.7 dB.

Tone levels (without ILDs and level roving) varied from 28 to 30 dB SL across trials for all participants but m2 and f7. For m2 and f7, tone levels on some trials were lowered to 25 and 22 dB SL, respectively, to limit the maximum presentation level to 95 dBA.

Order effects might have complicated comparisons of individual performance if the three ILD discrimination conditions had been run block-wise. Therefore, the conditions were interleaved on a trial-by-trial basis, that is, the three adaptive runs were performed at the same time. Since the imposed ILDs shifted both the second and fourth tones to the right side, participants were instructed to identify the interval in which both tones moved to the right (see Appendix A). Participants performed a short No Rove training run consisting of 6 trials followed by interleaved training runs of all three conditions of 10 trials each, before performing the three interleaved measurement runs of 50 trials each.

ILD discrimination performance as a function of log-transformed ILD was fitted using a Weibull PF model (Schütt et al., 2016), and the ILD JND was defined as the ILD corresponding to the 75 percent-correct point.

#### Intensity JND

The 2I-2AFC measurement of intensity JND (INT JND) at 500 Hz used sequences of diotic tones without any binaural cues. All tones in the reference interval had identical levels, and the same level was applied to the first and third tones of the target interval. The second and fourth tones of the target interval were presented at a higher level. The level increment relative to the other tones was varied adaptively. The set of possible level increments were 47 logarithmically scaled values from 0.1 to 10 dB, and all runs started with an increment of 6 dB. Tone levels (before application of the level increment) were 30 dB SL for all participants.

Participants were instructed to detect the interval in which the tones varied in loudness (see Appendix A). They performed a training run of 10 trials before performing the measurement run of 60 trials. Discrimination performance as a function of log-transformed level increment was fitted using a Weibull PF model (Schütt et al., 2016) and the INT JND was defined as the level increment corresponding to the 75 percent-correct point.

#### Apparatus

The psychoacoustic tests were performed in a sound-treated room at the Heuser Hearing Institute. All stimuli were generated in MATLAB at a sampling rate of 48 kHz, converted to analog signals using an RME Fireface UCX audio interface with 24-bit digital-to-analog conversion, and presented via Sennheiser HD 600 headphones. Stimulus presentation levels were calculated by adding sensation levels to interpolated audiometric thresholds (measured thresholds were interpolated in dB HL on a logarithmic frequency scale) and converting dB HL to dB sound pressure level in an artificial ear (GRAS 43AA) using the probe-tube method described in American National Standards Institute (2018), with a Sennheiser HDA 200 headphone and an Etymotic Research ER-7 C microphone used as reference standard earphone and probe-tube microphone, respectively.

#### Test sessions

Testing was conducted in two sessions of 1- to 2-hour duration each. After informed consent and audiological examination, the MoCA was administered. Testing then proceeded in the first session with four psychoacoustic tests in the following order: IPD FL, ILD JND, IPD JND, and IPD FL. Testing resumed in the second session with the following tests: IPD FL, INT JND, IPD JND, and IPD FL. Thus, to explore potential practice effects, the IPD FL was measured four times (each time preceded by ILD and IPD training as described above) and the IPD JND was measured twice. The second session concluded with the Trail Making Test. The time intervals between the first and second session varied across participants from a single day to 7 days (median of 3 days), with the exception of participant f9, who performed the second session 84 days after the first.

#### Statistical analysis

Analyses of covariance (ANCOVAs) were performed on general linear mixed-effects models (GLMMs; Bates, Maechler, Bolker, & Walker, 2019) with model selection via *F* tests with Kenward–Roger approximation for denominator degrees of freedom (Kuznetsova, Brockhoff, & Christensen, 2019). Least-squares means were calculated to estimate effect sizes and to perform post-hoc tests (Lenth, Love, & Hervé, 2019). The proportion of total variance explained by the fixed effects was quantified by computing the marginal *R*^2^ (Bartoń, 2018; Nakagawa & Schielzeth, 2013). Normality of residuals was judged by means of Quantile-Quantile plots. Where applicable, *p* values were corrected for multiple testing using the method by Benjamini and Yekutieli (2001), yielding the *q* value as the analog of the adjusted *p* value. The descriptors of correlation strength follow Evans (1996). The abbreviation *SD* stands for standard deviation.

## Results

### Cognitive Tests

#### MoCA

The MoCA scores for all participants are listed in Table 1. The mean score was 26.0 (*SD* = 2.2). Three of the 20 participants (f9, f17, and m20) failed to successfully draw the alternating trail without (uncorrected) errors in the untimed TMB subtask of the MoCA.

#### Trail Making Test

All participants completed the timed TMA and TMB tests in less than 300 seconds. The average TMA and TMB times were 38 (*SD* = 20) seconds and 75 (*SD* = 40) seconds, respectively. Individual TMA and TMB times ranged from 17 to 88 seconds and 31 to 160 seconds, respectively. The difference score TMB–TMA had an average value of 37 (*SD* = 25) and ranged from 4 to 89 seconds.

### Psychoacoustic Tests

#### IPD discrimination upper FL

**Figure 1.**
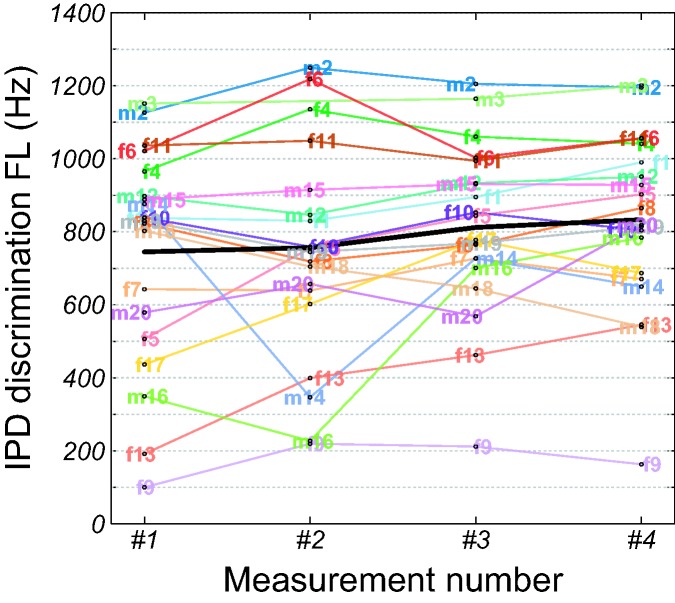
Individual IPD discrimination FLs as a function of measurement number. The thick black curve shows the least-squares means. IPD = interaural phase difference; FL = frequency limit.

Figure 1 shows individual IPD discrimination FLs as a function of measurement number. The data are given on a linear ordinate scale, as the residuals of a model of the log-transformed IPD FLs deviated from normality. Due to lack of time, participant m3 did not perform the second IPD FL measurement in the first session. Therefore, only three measurements are available for him. Participant f9 performed poorly in her first measurement, with a fitted IPD FL of 62 Hz. As this fit was based on extrapolation below the lowest presented frequency of 125 Hz, we replaced her IPD FL with the estimated upper bound of 100 Hz (see simulation of near-chance performance in the “Methods” section). The performance of participants f5, f9, f13, m16, and f17 were relatively poor in their first measurements but seemed to improve with time. Similarly, participant f1’s performance also seemed to improve. A GLMM of the IPD FLs confirmed that the effect of measurement number was significant, *F*(3, 56) = 2.98, *p* = .04, with average performance continuously improving (measurement 1: 745 Hz, measurement 2: 756 Hz, measurement 3: 812 Hz, measurement 4: 833 Hz). Post hoc tests revealed significant differences between measurements 1 and 4 as well as between measurements 2 and 4.

#### Interaural phase difference JND

**Figure 2.**
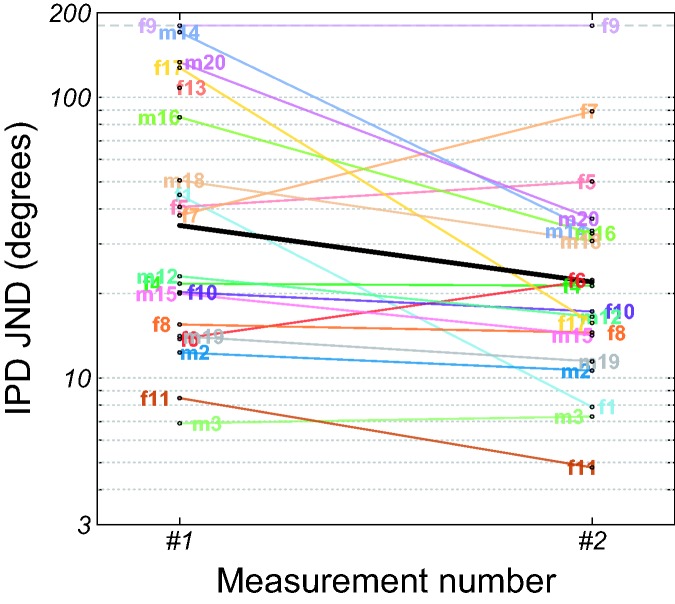
Individual IPD JNDs as a function of measurement number. The thick black curve shows the least-squares means. The dashed gray curve represents the maximum IPD of 180°. IPD = interaural phase difference; JND = just-noticeable difference.

Figure 2 shows individual IPD JNDs as a function of measurement number. Due to an oversight, participant f13 only performed a single IPD JND measurement. Participant f9 performed poorly in both measurements. Her fitted IPD JNDs exceeded the maximum presented IPD of 180° and were substituted by that value in the figure and in the subsequent statistical analyses. Her poor IPD discrimination performance at 500 Hz were consistent with her low IPD FLs, which indicated that she could discriminate between 0° and 180° IPDs only up to 220 Hz. Overall, the effect of measurement number was significant, GLMM of the log-transformed IPD JNDs: *F*(1, 18) = 6.82, *p* = .02, with a lower average IPD JND in the second measurement (22°) than in the first measurement (35°).

#### Interaural level difference JND

**Figure 3.**
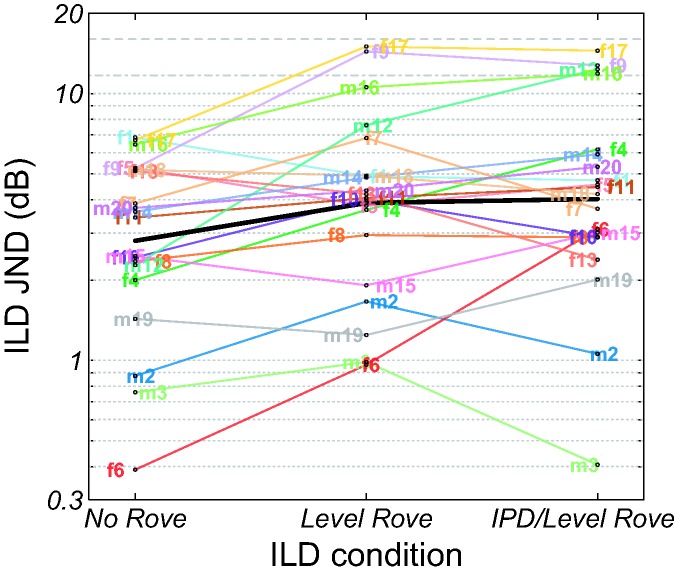
Individual ILD JNDs for the measurement conditions: No Rove, Level Rove, and IPD/Level Rove. The thick black curve shows the means. The dashed gray curve represents the maximum presented ILD of 16 dB. The dash-dotted gray curve represents the ILD of 11.7 dB above which the task could be accomplished based on single-ear cues alone. IPD = interaural phase difference; JND = just-noticeable difference; ILD = interaural level difference.

Individual ILD JNDs for the three measurement conditions No Rove, Level Rove, and IPD/Level Rove are shown in Figure 3. ILD JNDs are given on a logarithmic scale as the nontransformed JNDs (in dB) produced non-normal model residuals (cf. Spencer et al., 2016). All participants were able to perform the ILD discrimination task, yielding ILD JNDs smaller than 16 dB for all of the conditions. However, participants f9, m12, m16, and f17 showed markedly higher ILD JNDs in the Level Rove and IPD/Level Rove conditions than in the No Rove condition. It is possible that these participants used single-ear loudness cues in the No Rove condition. Furthermore, since their ILD JNDs in the roved conditions approached or exceeded the threshold ILD of 11.7 dB beyond which the task could be accomplished based on single-ear cues alone, it is possible that they did not use any spatial cues. A GLMM of the log-transformed ILD JNDs (log [ILD JND in dB]) confirmed the significance of measurement condition, *F*(2, 38) = 5.12, *p* = .01, with significantly lower ILD JNDs (*p* < .05) in the No Rove condition (2.8 dB) than in the Level Rove condition (3.9 dB) and IPD/Level Rove condition (4.0 dB). However, measurement condition was no longer significant when the ILD JNDs of participants f9, m12, m16, and f17 were excluded from the model, *F*(2, 30) = 1.43, *p* = .25. In this case, the mean ILD JNDs were 2.5, 3.0, and 3.0 dB for the No Rove, Level Rove, and IPD/Level Rove conditions, respectively.

#### Intensity JND

The average INT JND was 1.2 (*SD* = 0.6) dB. All participants showed similar performance without any outliers, as reflected by the small standard deviation. Individual INT JNDs ranged from 0.2 to 2.5 dB.

### Relationships Between Test Results

**Figure 4.**
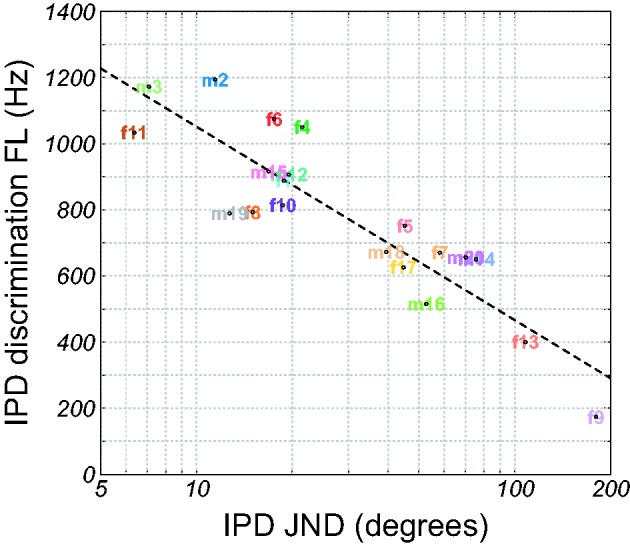
IPD discrimination FL as a function of the IPD JND at 500 Hz. The dashed curve shows a linear regression line. IPD = interaural phase difference; JND = just-noticeable difference; FL = frequency limit.

**Figure 5.**
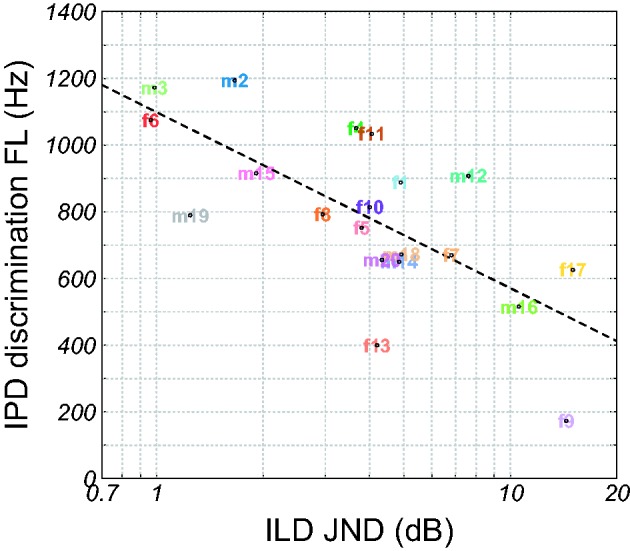
IPD discrimination FL as a function of the ILD JND at 500 Hz. The dashed curve shows a linear regression line. IPD = interaural phase difference; JND = just-noticeable difference; ILD = interaural level difference; FL = frequency limit.

To assess the strength of association between the various cognitive and psychoacoustic measures, we calculated Pearson’s product–moment correlation coefficients. The included variables were participant age, PTA_LF_, MoCA score, TMA time, TMB time, TMB–TMA, IPD FL and IPD JND, both averaged across repetitions, ILD JND in the Level Rove condition, and INT JND. We included the ILD JND in the Level Rove condition because single-ear cues could have been used in the No Rove condition, and the added random IPDs in the IPD/Level Rove condition could have affected individual performances in various ways (e.g., some participants might have been insensitive to added random IPDs, while others might have been distracted by them). Thus, the ILD JND in the Level Rove condition was the best measure of *spatial* discrimination based on binaural ILD cues. Log-transformed, normally distributed TMA and TMB times, IPD JNDs, ILD JNDs, and INT JNDs were used in the computation of the correlation coefficients. The resulting correlation matrix is listed in Table 2. After multiple testing correction (Benjamini & Yekutieli, 2001), all spatial psychoacoustic measures (IPD FL, IPD JND, and ILD JND) were significantly correlated. In addition, all of these spatial measures significantly correlated with Trail Making performance (TMB and/or TMA). The correlations remained significant when age and PTA_LF_ were partialled out (Table 3). Figure 4 shows the correlation between IPD FL and IPD JND, and Figure 5 shows the correlation between IPD FL and ILD JND.

**Table 2.** Pearson’s Product–Moment Correlation Coefficients Between Participant Age, PTA_LF_, MoCA Score, TMA Time, TMB Time, TMB–TMA, IPD FL, and IPD JND, Both Averaged Across Repetitions, ILD JND in the Level Rove Condition, and INT JND.

	PTA_LF_	MoCA	TMA	TMB	TMB–TMA	IPD FL	IPD JND	ILD JND	INT JND
Age	−.48†	−.36	.37	.60††	.61††	−.47†	.37	.23	−.20
PTA_LF_		.28	−.32	−.39	−.37	.18	−.20	−.21	.16
MoCA			−.34	−.51†	−.42	.50†	−.46†	−.53†	.09
TMA				.75**	.51†	−.72**	.66*	.74**	.26
TMB					.93***	−.86***	.81***	.61††	.09
TMB–TMA						−.76**	.72**	.41	.03
IPD FL							−.89***	−.70*	−.13
IPD JND								.66*	.03
ILD JND									−.01

*Note*. Values in boldface indicate significant correlations after multiple testing correction (Benjamini & Yekutieli, 2001), with correlations marked by *, **, or ***significant at *q* < .05, .01, or .001, respectively. Values marked by † indicate significant correlations before multiple testing correction, with † or †† significant at *p* < .05 or .01, respectively. IPD = interaural phase difference; JND = just-noticeable difference; ILD = interaural level difference; FL = frequency limit; MoCA = Montreal Cognitive Assessment; TMA = Trail Making Test part A; TMB = Trail Making Test part B; PTA_LF_ = low-frequency pure-tone thresholds average.

**Table 3.** Pearson’s Product–Moment Correlation Coefficients for Same Variables as in Table 2 But With Age and PTA_LF_ Partialled Out.

	TMA	TMB	TMB–TMA	IPD FL	IPD JND	ILD JND	INT JND
MoCA	−.22	−.38	−.26	.42	−.38	−.48†	.01
TMA		.70*	.37	−.69*	.61††	.72*	.39
TMB			.89***	−.83***	.79**	.60††	.29
TMB–TMA				−.68*	.67*	.34	.21
IPD FL					−.88***	−.71*	−.26
IPD JND						.64*	.12
ILD JND							.05

*Note.* Values in boldface indicate significant correlations after multiple testing correction (Benjamini & Yekutieli, 2001), with correlations marked by *, **, or ***significant at q<.05, .01, or .001, respectively. Values marked by † indicate significant correlations before multiple testing correction, with † or †† significant at p<.05 or .01, respectively.

IPD = interaural phase difference; JND = just-noticeable difference; ILD = interaural level difference; FL = frequency limit; MoCA = Montreal Cognitive Assessment; TMA = Trail Making Test part A; TMB = Trail Making Test part B; PTA_LF_ = low-frequency pure-tone thresholds average.

The following subsections present statistical models of the psychoacoustic variables IPD FL, IPD JND, and ILD JND. Given the small interindividual variability in INT JNDs, we do not present a statistical model of INT JND.

#### Model of IPD discrimination upper FL

**Figure 6.**
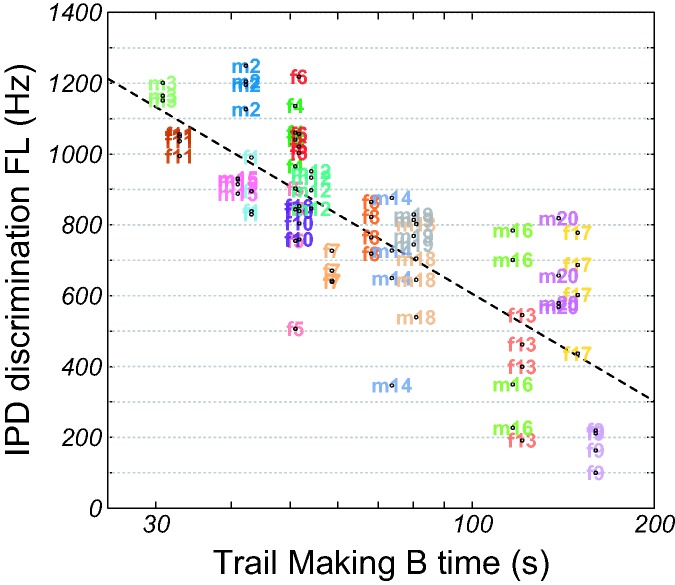
Individual IPD discrimination FLs as a function of TMB time. The dashed curve shows a linear regression line. IPD = interaural phase difference; FL = frequency limit.

To explore significant predictors of IPD FL, we performed ANCOVAs with IPD FL as the dependent variable. The tested covariates, in addition to measurement number, were age, PTA_LF_, MoCA score, log-transformed TMA and TMB times as well as the difference score TMB–TMA. We also tested the significance of presence of tinnitus and years of musical training. All predictors were tested in backward elimination. In addition to the significant effect of measurement number, *F*(3, 56) = 2.96, *p* = .04, TMB time was a significant predictor, *F*(1, 18) = 48.5, *p* < .0001. The interaction of TMB time with measurement number was not significant (*p* = .28). Figure 6 shows individual IPD FLs as a function of TMB time along with a regression line. IPD FL decreased with increasing TMB time. TMA time and the difference score TMB–TMA were not significant when TMB time was included in the model (*p* > .3). They were, however, both significant (*p* < .01) when TMB time was deliberately excluded. Effects of age, PTA_LF_, MoCA score, tinnitus, and musical training were not significant (*p* > .17). Together, the fixed effects measurement number and TMB time explained 63% of the variance.

To explore the extent to which TMB time mediated effects of age on IPD FL, we examined a path model with age as an independent variable, IPD FL as the dependent variable, and TMB time as the mediator (Baron & Kenny, 1986). The model revealed that age had no significant effect when TMB time was controlled, thus demonstrating perfect mediation of age by TMB time (Baron & Kenny, 1986).

#### Model of interaural phase difference JND

We performed ANCOVAs with IPD JND as the dependent variable and tested the same covariates as in the preceding subsection. In addition to the significant effect of measurement number, *F*(1, 18) = 5.67, *p* = .03, TMB time was a significant predictor, *F*(1, 18) = 31.9, *p* < .0001. IPD JNDs increased with increasing TMB time. The interaction with measurement number was not significant (*p* = .15). Similar to the IPD FL results, TMA time and the difference score TMB–TMA were not significant in the model with TMB time (*p* > .4), but were both significant (*p* < .05) when TMB time was deliberately excluded from the model. Effects of age, PTA_LF_, MoCA score, tinnitus, and musical training were not significant (*p* > .3). The fixed effects measurement number and TMB time explained 54% of the variance.

#### Model of interaural level difference JND

ANCOVAs with ILD JND as the dependent variable revealed significant effects of measurement condition, *F*(2, 38) = 5.12, *p* = .01, MoCA score, *F*(1, 17) = 7.28, *p* = .02, and TMA time, *F*(1, 17) = 13.8, *p* = .002. ILD JND increased with increasing TMA time and decreased with increasing MoCA score. Interaction terms were not significant (*p* > .5). Furthermore, TMB time was not significant (*p* = .81) when TMA time was included in the model but was significant (*p* = .004) when TMA time was deliberately excluded. The difference score TMB–TMA was not significant, regardless of whether TMA time was included or not (*p* > .3). Furthermore, effects of age, PTA_LF_, tinnitus, and musical training were not significant (*p* > .14). The fixed effects measurement condition, TMA time, and MoCA score together explained 54% of the variance.

## Discussion

Overall, the results of the individual cognitive and psychoacoustical tests reported in this work are consistent with the literature. Details of this consistency are discussed in the subsections that follow. The practice effects observed for some listeners in the IPD discrimination tasks are also discussed. Most important, however, were the clear and consistent relationships observed between primary measures, including IPD discrimination, ILD discrimination, and Trail Making. These relationships suggest that in an older HI population, IPD discrimination performance does not reflect TFS sensitivity per se, but must be influenced by factors that are not specifically auditory and may be of higher order. These possibilities are discussed with reference to a conceptual model.

### Cognitive Tests

The MoCA scores of our study participants were very similar in terms of mean and standard deviation to those reported in previous studies with HI participants (Dupuis, Marchuk, & Pichora-Fuller, 2016; Lin et al., 2017).

The Trail Making Test scores obtained in our study cannot easily be compared in absolute terms with the literature since we used a touchscreen version of the Trail Making Test, with identical but mirrored trails for part A and B. Nevertheless, the observed mean TMA and TMB times were similar to those reported by Woods et al. (2013) for participants with comparable hearing losses and those for the elderly, healthy participants in Tombaugh (2004) and Periáñez et al. (2007). Furthermore, our Trail Making scores were consistent with the literature in that TMB times were larger than TMA times by a factor of two, and TMB times correlated significantly with age and TMA times, as well as the difference score TMB–TMA (Arbuthnott & Frank, 2000; Corrigan & Hinkeldey, 1987; Tombaugh, 2004; Yeudall, Reddon, Gill, & Stefanyk, 1987).

### Psychoacoustic Tests

The mean IPD discrimination FL of 787 (*SD* = 257) Hz observed in this study was consistent with the mean IPD FLs ranging from 583 to 808 Hz in previous studies that used similar tone sequence stimuli (Füllgrabe & Moore, 2017; Moore & Sek, 2016; Neher et al., 2011).^1^ The average IPD JND of 28° observed at 500 Hz in our study was consistent with the average IPD JNDs of 25°, 25°, and 32° obtained by Hopkins and Moore (2011), Neher et al. (2012), and King et al. (2014), respectively, but smaller than the average IPD JND of 58° reported by Moore and Sek (2016).^2^ To our knowledge, ILD JNDs have not been measured previously in HI listeners with tone sequence stimuli such as those used in this study. However, the range of observed ILD JNDs in the Level Rove condition was consistent with the ILD discrimination thresholds for 1/3-octave noise bands centered at 500 Hz reported by Koehnke et al. (1995, Figure 3) and Spencer et al. (2016) for their HI participants.

#### Effects of practice on IPD discrimination

We observed significant performance improvements from measurement to measurement for IPD FLs and IPD JNDs. These improvements were consistent with the practice effects observed for ITD discrimination at 500 Hz by Spencer et al. (2016) in HI listeners and by Ortiz and Wright (2009, 2010) in NH listeners. In particular, for the IPD FLs the largest average performance improvement was observed between the two test sessions (between measurements 2 and 3). This is consistent with Ortiz and Wright (2010), whose NH participants reached their best ITD discrimination performance not immediately following training but with 10 hours of rest after training and into the following day. Presumably, new IPD discrimination skills had been acquired in the short-term during the first test session of this study, and consolidation of learning took place in the hours following and into the second test session. Füllgrabe et al. (2017) and Füllgrabe and Moore (2017) observed no practice effects for IPD FLs obtained with young and older NH listeners, respectively. Similarly, Hopkins and Moore (2010) observed no practice effects for IPD JNDs obtained with young NH listeners. However, the present results suggest that these findings do not transfer to HI listeners. Some of our HI participants improved with practice in both IPD FL and IPD JND measurements, resulting in average practice effects for the whole group. In comparison with the above mentioned studies, which repeatedly measured only IPD FLs (or IPD JNDs), we administered IPD JND and ILD JND measurements in between IPD FL measurements. This might have enhanced practice effects. However, it is clear from the present results that some participants did not reach asymptotic performance in the first two or three measurement runs. Thus, there is no reason to believe that practice effects would have been altogether absent if only IPD FLs (or IPD JNDs) had been measured.

In this study, we adopted a training paradigm that has been used in many previous studies exploring IPD discrimination with tone sequence stimuli (e.g., Bianchi et al., 2019; Füllgrabe et al., 2017; Hopkins & Moore, 2011; Neher et al., 2011): IPD cues were initially replaced with ILD cues, because ILD cues presumably result in more discriminable lateralization for all participants, in particular for older HI listeners. However, Ortiz and Wright (2010) found that, in order to improve NH listeners’ ITD discrimination performance, training based on ITD discrimination proved more effective than ILD discrimination. It is unclear if this would hold true for older HI listeners. However, considering that ILD stimuli can introduce listeners to single-ear loudness cues, it might be better to resort to training listeners only with IPD stimuli in preparation for IPD discrimination measurements.

### Relationships Between Test Results

A moderate negative correlation (*r* = −.47) was observed between IPD FL and age, which was no longer significant when the multiple testing correction was applied. Furthermore, the correlation between IPD JND and age (*r* = .37) was not significant, and no significant effect of age was observed in any of the statistical models. Although low power contributed to the lack of significance (the sample size of 20 participants resulted in a power of 0.8 for detecting associations of *r* = .58 or stronger), the strength of the observed age correlations was nevertheless consistent with previous studies that measured IPD FLs and IPD JNDs in older listeners with and without low-frequency hearing losses (Füllgrabe et al., 2018; King et al., 2014; Ross et al., 2007a). However, a wide range of age correlations has been reported. Some studies observed very weak correlations of age with IPD FLs (Füllgrabe & Moore, 2017; Moore & Sek, 2016), while others have observed strong correlations with IPD JNDs (Füllgrabe & Moore, 2018; Hopkins & Moore, 2011; Moore, Glasberg, Stoev, Füllgrabe, & Hopkins, 2012).

The correlations with PTA_LF_ were weak and not significant, and PTA_LF_ was not a significant predictor in any of the statistical models either. This can be attributed to the homogeneity of our participant group in terms of their audiograms (best and worst PTA_LF_ differed only by 15 dB, with an *SD* of 4.5 dB across listeners) as well as low power. Nevertheless, the observed weak strength of correlations was consistent with previous reports of weak correlations between IPD FL, IPD JND, ILD JND, and pure-tone thresholds or PTA_LF_ (Füllgrabe et al., 2018; Hopkins & Moore, 2011; Spencer et al., 2016). However, moderate correlations between IPD FLs, IPD JNDs, and hearing loss have also been reported (Füllgrabe & Moore, 2017; Füllgrabe & Moore, 2018; King et al., 2014; Moore & Sek, 2016).

Bianchi et al. (2019) observed that both NH and HI musicians with at least 8 years of formal music education showed lower IPD FLs than nonmusicians. Our data showed no significant effect of years of musical training, which may be attributable to low power as only six of our participants had 4 years or more of musical training. Furthermore, we observed no significant effect of tinnitus on spatial discrimination, which is consistent with Hyvärinen, Mendonça, Santala, Pulkki, and Aarnisalo (2016).

IPD FL was very strongly correlated with IPD JND (*r* = −.89), reflecting the similarity of these measures in assessing IPD discrimination (Füllgrabe et al., 2017; Moore & Sek, 2016). Both IPD FL and IPD JND were very strongly correlated with TMB time (*r* = −.86 and *r* = .81, respectively), and TMB time was also the strongest predictor in the respective statistical models. Besides TMB time, significant correlations were also observed with TMA time and the difference score TMB–TMA (with absolute values of *r* ranging from .66 to .76). Interestingly, ILD JND was also significantly correlated with TMA time (*r* = .74), IPD FL (*r* = −.70), and IPD JND (*r* = .66). In addition to TMA time, statistical modeling showed that the MoCA score was also a significant predictor of ILD JND.

Taken together, the observed significant relationships between IPD discrimination, ILD discrimination, and Trail Making formed a closed triangle of associations. These associations remained mostly unchanged when effects of age and hearing loss were partialled out. Füllgrabe et al. (2015) previously observed a moderate association between IPD discrimination and TMB time in older listeners with normal pure-tone thresholds in the low frequencies. Furthermore, three previous studies observed significant associations between IPD or ITD JNDs and ILD JNDs. Ochi et al. (2016) observed a moderate correlation between ITD JND and ILD JND for complex tones centered at 1100 Hz in a mixed group of young NH and older NH and HI listeners. Whiteford et al. (2017) also found a moderate correlation between JNDs for discrimination of dynamic IPDs and dynamic ILDs for 500-Hz tones in a sample comprising young and older listeners with normal hearing in the low frequencies. Spencer et al. (2016) observed a very strong correlation between IPD JND and ILD JND for 1/3-octave noise bands at 500 Hz in HI listeners.

IPD or ITD processing in the auditory system is based on phase locking of auditory nerve action potentials to TFS and subsequent coincidence detection in the auditory brainstem (McAlpine, 2005; Young & Oertel, 2004), whereas ILDs are thought to be encoded in the brainstem independently of TFS (Brown & Tollin, 2016; Franken, Joris, & Smith, 2018). Thus, if interindividual variability in IPD discrimination performance reflected deficits specific to TFS processing, which is not utilized in ILD coding, IPD and ILD discrimination performance would not be associated. Further, if variability in IPD discrimination performance reflected modality-specific deficits in audition as opposed to modality-general deficits, IPD discrimination would not be associated with a nonauditory cognitive measure such as Trail Making. Therefore, the associations between IPD and ILD discrimination observed here (and in three previous studies) and the observed associations between IPD discrimination and Trail Making imply that the variability in IPD discrimination performance across HI listeners did not exclusively reflect deficits specific to TFS processing or even audition in general.

Previous studies have explored relationships between TFS processing and cognitive functions in HI participants (Neher et al., 2011, 2012; Rönnberg et al., 2016) and older participants with normal pure-tone thresholds in the low frequencies (Füllgrabe et al., 2015, 2018). Rönnberg et al. (2016) reported the outcomes of exploratory factor analyses performed on a test battery of various auditory and cognitive tests completed by 200 HI participants. They observed a significant correlation between a TFS factor and a cognition factor. The TFS factor loaded primarily on IPD JNDs measured at 250 Hz and to some extent on monaural spectro-temporal modulation thresholds (Bernstein et al., 2013), while the cognition factor loaded primarily on measures of executive function and secondly on auditory working memory. Rönnberg et al. (2016) speculated that one possible interpretation for the observed relationship between TFS and cognition factors was that both IPD JND and spectro-temporal modulation detection tasks placed high demands on auditory working memory. However, this is not supported by Füllgrabe et al. (2015), who observed no significant correlations between TFS processing abilities and working memory capacity assessed in terms of reading span. In that study, the cognitive measures that correlated significantly with TFS sensitivity were TMB, a map search test, a digits forward test, an auditory elevator counting test, and a block design test (for details, see Füllgrabe et al., 2015). Both Füllgrabe et al. (2015) and Füllgrabe et al. (2018) observed no significant correlations between TFS sensitivity and performance on a matrix reasoning test, which is a measure of nonverbal intelligence without imposed time limits. Consistent with Füllgrabe et al. (2015), Neher et al. (2012) observed a significant correlation between IPD JND and map-search performance but not reading span. Neher et al. (2011) also observed no correlation between IPD FL and reading span. Füllgrabe et al. (2015) used a composite TFS score consisting of both monaural and binaural TFS measures in their correlational analysis. When considered separately, performance on the binaural IPD discrimination task correlated more strongly with all of the above mentioned cognitive measures than performance on the monaural frequency discrimination task using complex tones (Füllgrabe et al., 2015, supplementary material). However, the significance of these differences in correlations was not assessed, and thus no firm conclusions can be drawn as to whether IPD discrimination primarily drove the significant correlations between the cognitive measures and the composite TFS score or not. Nevertheless, at least the studies by Neher et al. (2012) and Rönnberg et al. (2016) who observed significant correlations between cognitive abilities and TFS sensitivity could alternatively be interpreted as indicating a relationship between cognitive abilities and spatial auditory function (binaural processing ability in terms of IPD discrimination) instead of basic, monaural TFS processing abilities.

TMA time, TMB time, and the difference score TMB–TMA are partly complementary measures of cognitive abilities (e.g., Arbuthnott & Frank, 2000; Bowie & Harvey, 2006; Sánchez-Cubillo et al., 2009). TMA time indexes processing speed, visual search, and motor skills. In addition to these factors, TMB time reflects executive function including the ability to manipulate information in working memory and attentional control processes necessary to manage rapid task switching (e.g., Arbuthnott & Frank, 2000; Sánchez-Cubillo et al., 2009). As regards the interpretation of the difference score TMB–TMA, no consensus has been reached. Sánchez-Cubillo et al. (2009) considered it to be a relatively pure indicator of executive function, whereas Arbuthnott and Frank (2000) suggested that it reflected processing speed and visual search. In this study, IPD discrimination was correlated with and predicted by TMA time, TMB time, and the difference score TMB–TMA. Thus, it seems that processing speed, visual search, and executive function may have played a role in the observed relationships with IPD discrimination. ILD discrimination was significantly correlated with TMA time but not TMB time or the difference TMB–TMA. It remains unclear why executive function would have been linked with IPD discrimination but not ILD discrimination. In addition to processing speed, visual search, and executive function, the Trail Making Test indexed motor speed. However, since motor speed should not have influenced IPD or ILD discrimination performance, it is not discussed further here.

**Figure 7.**
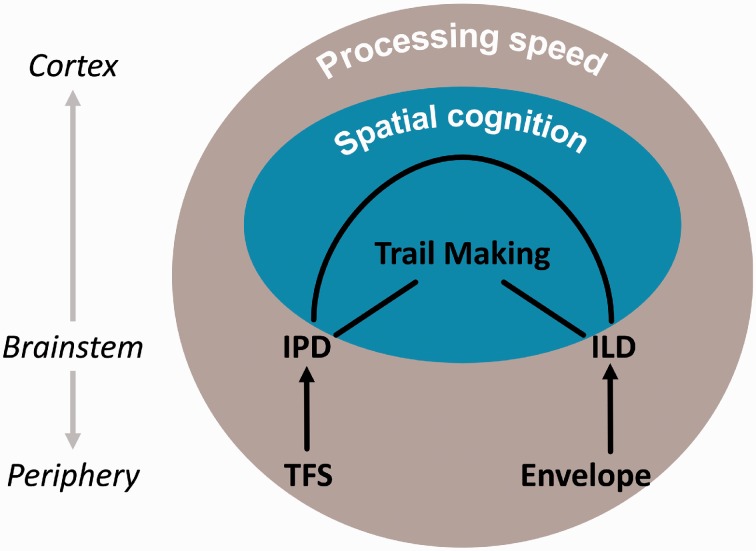
Conceptual model of relationships between IPD sensitivity, ILD sensitivity, and Trail Making. IPD = interaural phase difference; ILD = interaural level difference; TFS = temporal fine structure.

It is possible that the observed relationships between IPD discrimination, ILD discrimination, and Trail Making times could be traced back to a common cause (Humes & Young, 2016; Pichora-Fuller, 2003), as visualized in Figure 7. Such a common cause could be individual differences in processing speed, which may arise on a peripheral neural level and/or on a global cognitive level (Figure 7). One possible cause for individual differences in processing speed is global slowing with aging (Myerson, Hale, Wagstaff, Poon, & Smith, 1990; Salthouse, 1996; Schneider, Pichora-Fuller, & Daneman, 2010). Indeed, a path model revealed that TMB time was a perfect mediator of age-related effects on IPD FL in this study. Thus, age had no effect on IPD FL when TMB time was controlled. However, we observed no significant correlation between ILD JND and age, which is consistent with earlier reports that brainstem ILD coding is resilient to aging (Caspary, Ling, Turner, & Hughes, 2008). Thus, age was likely not the sole determinant of the observed relationships between spatial audition and cognition in this study. This is in agreement with the conclusion by Humes and Young (2016) that sensory processing and cognition are linked independently of age. Importantly, this does not rule out the possibility that individual differences in processing speed, independent of age, were responsible for the observed correlations. Consistent with this hypothesis, Oberfeld and Klöckner-Nowotny (2016) observed a significant correlation between IPD JND and processing speed, as indexed by response time in the neutral condition of a visual flanker task, in a group of young NH listeners.

The IPD and ILD discrimination tasks were both spatial tasks, and the Trail Making Test parts A and B involved the processing of spatial information required for visual search. Thus, the mutual relationships between these measures might indicate some kind of modality-general spatial processing deficit as a common cause. Indeed, all of our participants performed well on the diotic intensity discrimination task as opposed to the spatial discrimination tasks. Similarly, Füllgrabe et al. (2015) observed no significant correlations between amplitude-modulation detection thresholds for diotic tones and cognitive abilities after applying a multiple-testing correction. If a spatial processing deficit explained the observed relationships between IPD and ILD discrimination performance, it would need to result from processes beyond the level of the brainstem, because (a) IPDs and ILDs are encoded independently at the level of the mid-brain (Brown & Tollin, 2016), and (b) our data show that the relationship between IPD and ILD discrimination performance was perfectly mediated by Trail Making performance. The hypotheses of a spatial processing deficit and individual differences in processing speed are not mutually exclusive. A modality-general spatial processing deficit could conceivably arise in conjunction with slowed processing speed (see Figure 7).

One might argue that the difference score TMB–TMA reflected neither processing speed nor visual search, since these factors were common to both TMA and TMB tasks. Thus, the observed correlation between TMB–TMA and IPD discrimination would seem at odds with the above hypotheses of a spatial processing deficit or individual differences in processing speed. However, such an interpretation of the difference score is not supported by Arbuthnott and Frank (2000), who suggested that the difference TMB–TMA did in fact reflect processing speed and visual search. The latter view is in agreement with the very strong correlation between TMB–TMA and TMB time observed in this study and in previous studies (Arbuthnott & Frank, 2000; Corrigan & Hinkeldey, 1987; Sánchez-Cubillo et al., 2009), taken together with the consensus that TMB time reflects visual search (see Table 1 in Sánchez-Cubillo et al., 2009).

One avenue to investigate the hypotheses of a spatial processing deficit or individual differences in processing speed in future studies would be to include more spatial and nonspatial psychoacoustic measures, for example, frequency-modulation detection (Strelcyk & Dau, 2009; Whiteford et al., 2017), and nonspatial cognitive tests of processing speed in addition to the Trail Making Test used here. We mentioned in the “Introduction” section that 4 out of 20 participants in a previous, unpublished study were not able to perform above chance on a similar IPD FL task. In contrast, all participants in this study were able to perform the task. This also highlights the necessity for follow-up research, possibly in a larger study sample. Importantly, the results of the unpublished study are consistent with the associations observed in this study: The three participants who failed on the IPD FL task and who returned for follow-up testing also failed on the ILD discrimination task in the Level Rove and IPD/Level Rove conditions, and two of them failed on the untimed TMB subtask of the MoCA.

One might speculate that the spatial auditory and cognitive tasks, in particular TMB, used in this study had in common a high task complexity that required sustained attention throughout task performance. This could have influenced both auditory and cognitive performance. However, such an explanation for the observed correlations does not seem to be consistent with the results of Füllgrabe et al. (2015, 2018) and Neher et al. (2011, 2012), who used complex reading span or matrix reasoning tests but did not observe significant correlations between IPD discrimination and reading span or matrix reasoning scores. In this regard, it would be valuable to employ objective measures of IPD sensitivity (e.g., Grose et al., 2017; Papesh et al., 2017; Ross et al, 2007a; Ross, Tremblay, & Picton, 2007b; Vercammen, Goossens, Undurraga, Wouters, & van Wieringen, 2018) in addition to psychoacoustic measures to further clarify relationships between spatial audition and cognition in HI individuals.

An important implication of the demonstrated relationship between IPD sensitivity and Trail Making performance is that results from a simple auditory test might be effectively used to predict performance in higher level spatial and/or cognitive domains (cf. Humes & Young, 2016). In this way, IPD sensitivity could potentially be used as a marker for other cognitive or processing speed deficits. A similar marker-based approach has already been proposed for Alzheimer’s disease, where correlates between disease subtypes and spatial hearing deficits have been observed (Golden et al., 2014).

## Conclusions

Strong to very strong correlations were observed between IPD discrimination, ILD discrimination, and Trail Making performance in a sample of 20 older HI listeners with matched moderate sloping to severe sensorineural hearing losses. These correlations imply that interindividual variability in IPD discrimination performance did not exclusively reflect deficits specific to TFS processing or even audition in general. Instead, a modality-general spatial processing deficit and/or individual differences in global processing speed may have affected both spatial auditory and cognitive performance. Significant performance improvements in IPD discrimination were also observed across test sessions.

## Appendix A—Participant Instructions

The instructions for the Trail Making Test were displayed to the participants on the touchscreen in front of them. The instructions for the psychoacoustic tasks were given to them in writing while being read out aloud by the experimenter.

### Instructions for Trail Making Test

#### Part A

On the next screen, you will see circles with numbers. Begin by tapping number 1, followed by 2, then 3, then 4, and so on, until you reach the end (1-2-3-4 …). When you tap the correct circle, a connecting line will be drawn. If you do not tap the correct circle, no line will be drawn. The test is timed. Work as fast as you can. When you are ready, tap the screen to begin a practice run.

#### Part B

On the next screen, you will see circles with numbers and letters. Begin by tapping number 1, followed by A, then 2, then B, then 3, then C, and so on, until you reach the end (1-A-2-B-3-C…). When you tap the correct circle, a connecting line will be drawn. If you do not tap the correct circle, no line will be drawn. The test is timed. Work as fast as you can. When you are ready, tap the screen to begin a practice run.

### Instructions for IPD Discrimination

In this measurement, we test how well you can hear certain differences between sounds. You will be listening to tones presented over headphones. On each trial, you will hear two tone sequences:

beep beep beep beep  beep beep beep beep

While they are playing, the first tone sequence and second tone sequence are visually highlighted on the touchscreen in front of you by the labels “1st” and “2nd,” respectively. Please take a look at the screen now.

The first and second tone sequences differ in terms of their *positions*. This is what you need to listen for: One tone sequence will be positioned unmoving in the center of your head. It sounds like mono presentation. In the other tone sequence, *movement* will occur: The tones will move repeatedly from the center to the side of your head. This may sound like going from mono to stereo. For example:

             beep   beep

beep beep beep beep  beep   beep

*Your task is to identify the tone sequence where the movement occurred.* Whether the movement will occur in the first or second tone sequence will vary randomly from trial to trial. If you heard movement in the first sequence …

  beep   beep

beep   beep      beep beep beep beep      Press “1st”

If you heard movement in the second sequence …

              beep    beep

beep beep beep beep   beep    beep        Press “2nd”

The task will vary in difficulty from trial to trial. If you are not able to tell whether movement occurred in the first or second tone sequence, just guess one of the two (“1st” or “2nd”).

After you enter your response, you will receive feedback: The correct response will be highlighted in green. Please pay attention to this feedback. We will begin with a training run.

### Instructions for ILD Discrimination

The next measurement is very similar to the preceding measurements. Again, you need to listen for movement in the tone sequences.

*Your task is to detect the tone sequence where both tones move to the right side.*

Note, the tones may vary in loudness. However, you cannot accomplish the task by focusing on the loudness of the tones because these are random loudness variations. Instead, *listen for movement* and indicate whether it was the first or second tone sequence in which both tones moved to the right side.

After you enter your response, you will receive feedback: The correct response will be highlighted in green. Please pay attention to this feedback. We will begin with a training run.

### Instructions for Intensity Discrimination

In the preceding measurements, you listened for movement. In contrast, in the next measurement you need to listen for changes in loudness.

*Your task is to detect the tone sequence in which the tones varied in loudness.* For example:

beep beep beep beep   beep BEEP beep BEEP      Press “2nd”

The task will vary in difficulty from trial to trial.

After you enter your response, you will receive feedback: The correct response will be highlighted in green. Please pay attention to this feedback. We will begin with a training run.

## Notes

1 Füllgrabe and Moore (2017), whose HI participants were on average 7 years older and had milder low-frequency losses (PTA_LF_) by 6 dB, obtained an (arithmetic) mean IPD FL of 763 (*SD* = 277) Hz. Moore and Sek (2016), whose listeners were on average 3 years older and had milder PTA_LF_’s by 6 dB, observed a mean IPD FL of 583 (*SD* = 290) Hz. The participants in Neher et al. (2011) had on average milder low-frequency losses by 15 dB compared with this study. When only considering their 10 participants with the strongest low-frequency losses, the difference in mean PTA_LF_ shrinks to 7 dB. These participants were on average 2 years younger than our participants, and their mean IPD FL was 808 (*SD* = 255) Hz.

2 Moore and Sek (2016) observed an average IPD JND of 58°. Excluding the 20 youngest participants and 29 participants with the mildest hearing losses at 500 Hz in King et al. (2014), the average IPD JND for the remaining 10 HI participants in that study was 32°. These 10 participants were on average 3 years younger and still had 17 dB milder losses at 500 Hz than those in this study. Neher et al. (2012) observed an average IPD JND of 25° for their nine HI participants aged 49 years and older (on average 6 years younger than those in this study) with low-frequency losses averaged across 125, 250, 500, and 750 Hz ranging from 19 to 43 dB HL (the corresponding low-frequency losses in this study ranged from 34 to 52 dB HL). Finally, excluding the 3 youngest participants and 12 participants with the mildest losses in Hopkins and Moore (2011), the remaining 10 HI participants showed an average IPD JND of 25°. These 10 participants were on average younger by 4 years than the participants in this study and also had milder losses at 500 Hz, on average by 14 dB.
